# Transcriptome and Proteome of Methicillin-Resistant Staphylococcus aureus Small-Colony Variants Reveal Changed Metabolism and Increased Immune Evasion

**DOI:** 10.1128/spectrum.01898-22

**Published:** 2023-02-14

**Authors:** Si Liu, Hongbin Chen, Juan Chen, Tianyi Wang, Shangyu Tu, Xiaoyang Zhang, Qi Wang, Yuyao Yin, Yawei Zhang, Xiaojuan Wang, Chunjiang Zhao, Hui Wang

**Affiliations:** a Department of Clinical Laboratory, Peking University People’s Hospital, Beijing, China; b Department of Clinical Laboratory, The Affiliated Qingdao Central Hospital of Qingdao University, Qingdao, Shandong, China; c Peking University Health Science Center, Beijing, China; University of Calgary

**Keywords:** MRSA, small-colony variants, metabolism, glycolysis, immune evasion

## Abstract

Methicillin-resistant Staphylococcus aureus (MRSA) infection has become a public health crisis. Recently, we isolated small-colony variants (SCVs) of MRSA, which are characterized by slow growth, decreased virulence, increased antibiotic resistance, and immune evasion. In the present study, we provided proteomic and transcriptomic profiles of clinical MRSA sequence type 239 (ST239) normal strains and SCVs and attempted to identify the key genes or pathways closely related to SCV formation and survival. RNAs and proteins were extracted and subjected to RNA sequencing and mass spectrometry, and the transcriptome and proteome were evaluated via bioinformatic analysis. The results were verified by functional assays. In total, 822 differentially expressed genes (DEGs) and 773 differentially expressed proteins (DEPs) were identified; of these, 286 DEGs and DEPs were correlated and subjected to Kyoto Encyclopedia Genes and Genomes analysis. Some pathways were significant, including ABC transporters, ribosome biogenesis, and metabolic pathways such as glycolysis/gluconeogenesis and the citrate cycle (tricarboxylic acid [TCA] cycle). Based on these results, we found that the downregulation of ABC transporters and the TCA cycle pathway resulted in electron transport chain deficiencies and reduced ATP production in SCVs, leading to a dependence on glycolysis and its upregulation. In addition, the upregulation of capsule polysaccharides and the downregulation of surface proteins prevented phagocytosis and reduced the adhesion of host cells, contributing to immune evasion by SCVs. These findings contribute to a better understanding of the mechanisms of SCV formation and survival.

**IMPORTANCE** Small-colony variants (SCVs) of Staphylococcus aureus have drawn increasing research attention. Owing to their slow growth, atypical colony morphology, and unusual metabolic characteristics, SCVs often cause confusion in the laboratory. Furthermore, clinical treatment of SCVs is challenging owing to their antibiotic resistance and immune evasion, leading to persistent and recurrent infections. However, the mechanisms underlying their formation remain unclear. In this study, we isolated SCVs of methicillin-resistant S. aureus and provided transcriptomic and proteomic profiles of normal strains and SCVs. Based on our analysis, glycolysis upregulation and TCA cycle downregulation affected the electron transport chain and energy supply, leading to slower metabolism. Moreover, capsular biosynthesis was increased, while the number of surface proteins decreased, thus promoting immune evasion by SCVs.

## INTRODUCTION

Staphylococcus aureus infection is a major cause of skin, soft tissue, respiratory, bone, joint, and endovascular disorders, which can lead to a diverse range of diseases such as bacteremia, endocarditis, sepsis, toxic shock syndrome, and metastatic infections (1). Its impact is enhanced by the development of antibiotic resistance; one of the most notable strains is methicillin-resistant S. aureus (MRSA), which is resistant to virtually all β-lactam antibiotics (2). MRSA, which was first reported in the 1960s (3), played an important role in the emergence and global spread of antibiotic-resistant bacteria. This strain has had a significant impact on clinical outcomes compared with methicillin-susceptible S. aureus (4).

Among MRSA isolates, small-colony variants (SCVs) have attracted increasing attention (5–7). S. aureus SCVs are typically characterized by low growth rates (8), atypical colony morphology (5), unusual metabolic characteristics (5), reduced electron transport chain activity (5, 8), decreased virulence factor production (8), and reduced susceptibility to antimicrobials (8), thus complicating identification in the laboratory and hindering clinical diagnoses. They are also marked by auxotrophs for menadione, hemin, and thymidine (9). Furthermore, differentially expressed small non-protein-coding RNAs have also been identified to play a role in the formation of the clinical SCV phenotype (10). Compared to their normal counterparts, SCVs survive more easily in host cells and protect themselves from the host immune response and antibiotic treatment (11), thus contributing to prolonged infections. However, the mechanism of clinical SCVs formation remains unclear.

Recently, with the rapid development of omics technology, research on transcriptomes and proteomes has elucidated new avenues for studying the expression differences of strains at the RNA and protein levels. In previous studies, researchers compared the differences between normal S. aureus strains and SCV phenotypes using transcriptomic and proteomic approaches to understand the mechanism by which SCVs form (12, 13). Additionally, proteomic and transcriptomic profiles were used to assess the relative abundances of different functional categories of S. aureus, contributing to the global understanding of biological systems (14). The results of single omics analyses may have limited applicability, whereas the results of two combined omics methods can be verified by comparing the results of each. Thus, transcriptomics and proteomics were combined in this study to analyze the differences between normal S. aureus strains and their mutated SCV strains.

In this study, the proteomic and transcriptomic profiles of SCV phenotype strain IE2 were compared to normal phenotype strain IE1 to determine key genes or pathways closely related to SCVs, contributing to a better understanding of the mechanisms underlying SCVs formation and survival.

## RESULTS

### Comparisons of IE1 and IE2 phenotypes and genotypes.

**FIG 1:**
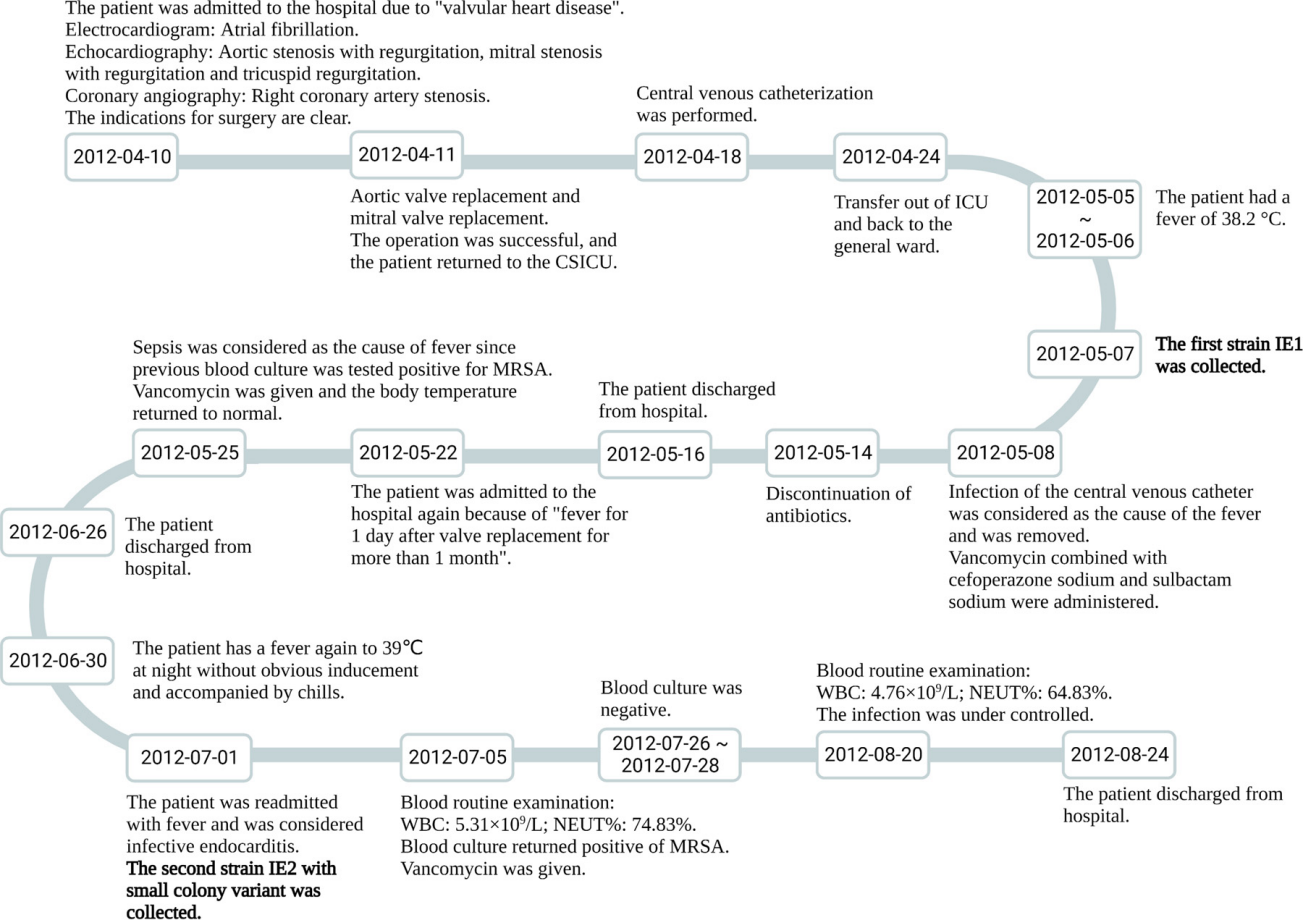
Timeline of disease progression of the patient. Strain collection is indicated in boldface type. WBC, white blood cell.

**FIG 2:**
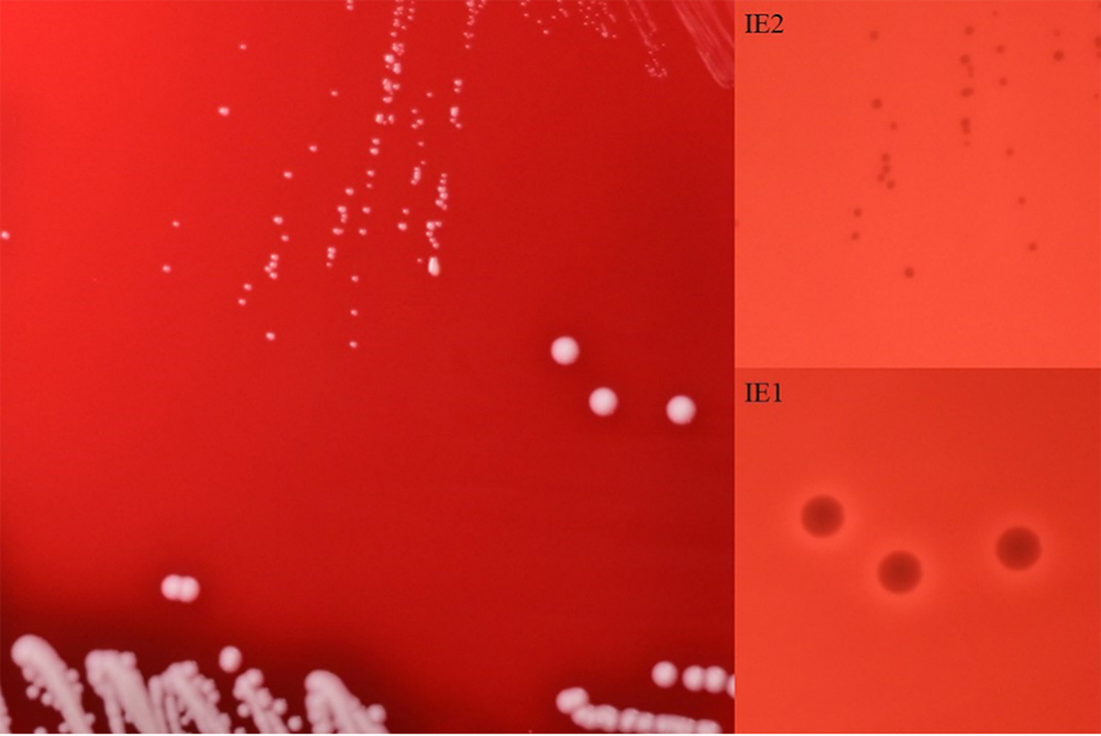
Normal strain IE1 and SCV strain IE2 were cultured on Columbia blood plate medium at 37°C for 24 h. The left picture was taken under normal light, and the right picture was taken with underplate lighting.

Normal strain IE1 and strain IE2 were isolated from the same patient during disease progression (Fig. 1). Their characteristics in Columbia blood plate medium were noted to exhibit significant differences. The colonies of IE2 showed a small morphology and no hemolytic rings (Fig. 2). Their whole genomes were analyzed, revealing that they are phylogenetically close (see Tables S1 and S2 and Fig. S1 in the supplemental material).

### Transcriptomic analysis of IE1 and IE2.

A total of 2,697 genes were identified in SCV strain IE2; compared with IE1, 460 genes were downregulated and 362 genes were upregulated in IE2 (Data Set S1). Cluster analysis of differentially expressed genes (DEGs) also showed that more genes were downregulated in IE2 than in IE1 (Fig. S2). In addition, among the DEGs, the 33 most significantly expressed genes with log_2_ (fold change) ≥ 5 or ≤ −5 and *P* values of <0.01 were filtered, including 11 downregulated genes and 22 upregulated genes (Data Set S1). Of these DEGs, genes encoding ATP-binding cassette (ABC) transporters, alpha-hemolysin, fibrinogen-binding protein, and YSIRK signal domain/LPXTG anchor domain surface protein were downregulated, whereas genes involved in capsular polysaccharide (CP) biosynthesis and urease subunits were upregulated. Relative gene expression was determined using real-time quantitative reverse transcription PCR (RT-qPCR) (Fig. S3).

**FIG 3:**
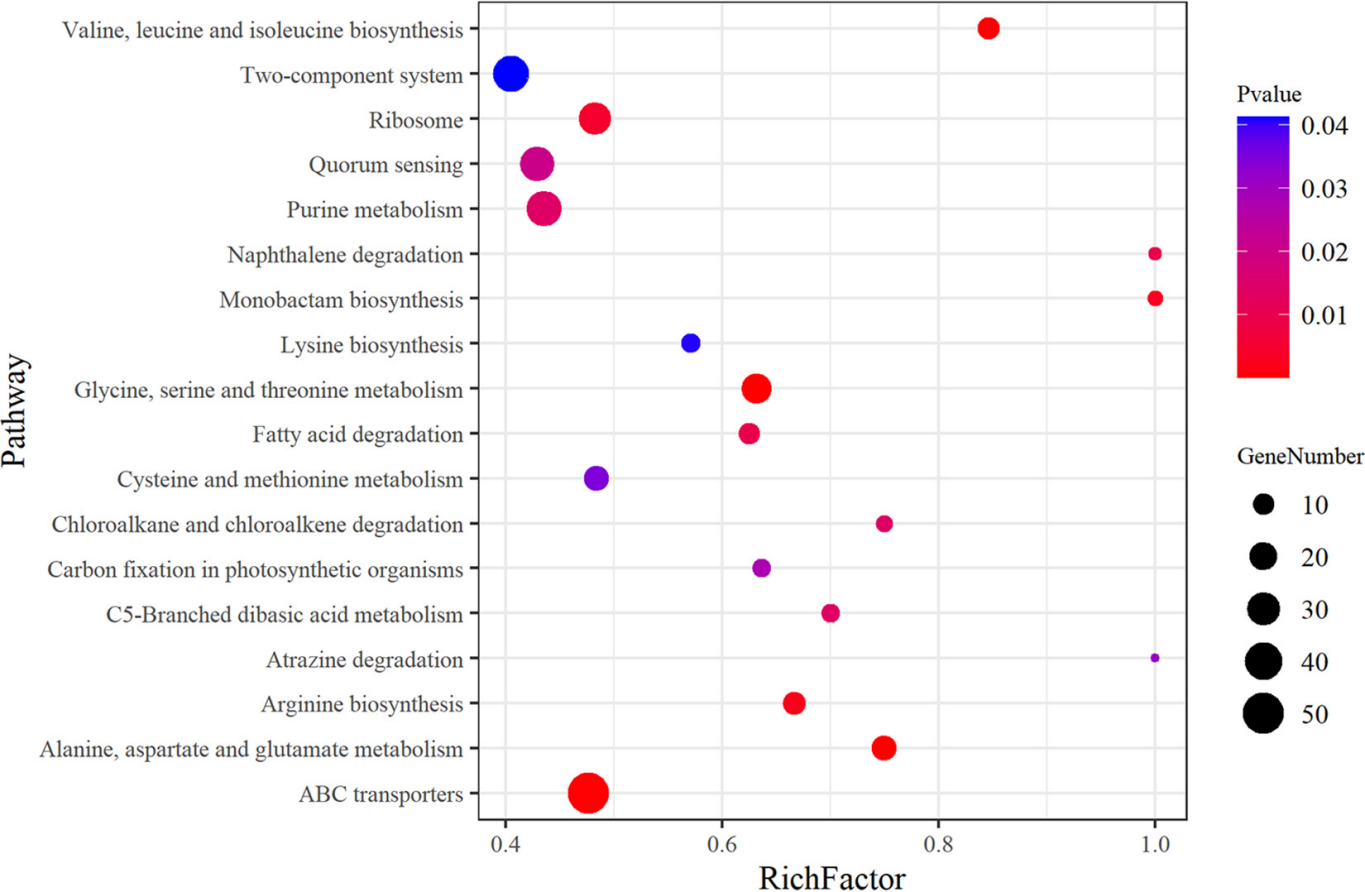
KEGG enrichment analysis of transcriptome DEGs of S. aureus SCV strain IE2 compared to normal strain IE1. RichFactor is the ratio between the number of DEGs and all genes with pathway annotations, indicating the proportion of DEGs in this pathway. The sizes of the dots denote the number of DEGs, and colors correspond to the *P* value range.

Based on Kyoto Encyclopedia of Genes and Genomes (KEGG) pathway analysis, a total of 651 DEGs were annotated with 108 pathways (Data Set S2), and the vast majority of the DEGs (447 genes) were involved in metabolism. To identify the most statistically significant pathways, we set a *P* value of <0.05, yielding 18 pathways (Fig. 3). Most of these genes were related to metabolism, especially amino acid metabolism, and altered metabolic characteristics were observed in the SCV strain. In addition, ABC transporters, quorum sensing, and two-component systems were found to play important roles in SCV formation (Fig. 3).

### Proteomic analysis of IE1 and IE2.

Totals of 21,308 peptides and 2,163 proteins were identified under the threshold of a false discovery rate (FDR of ≤0.01). Compared with normal strain IE1, SCV strain IE2 had 773 differentially expressed proteins (DEPs), of which 439 were upregulated and 334 were downregulated (Data Set S3). Moreover, we set a protein ratio of ≥4 or ≤0.25 and a *Q* value of <0.01 as criteria to filter the most statistically significantly expressed proteins. After screening, we obtained 29 proteins, of which 19 were downregulated and 10 were upregulated (Data Set S3). Moreover, protein expression was associated with ABC transporters such as the iron ABC transporter substrate-binding protein and a lantibiotic ABC transporter ATP-binding protein. In contrast, the expression of capsular polysaccharide biosynthesis proteins was upregulated, suggesting that SCV development is closely related to immune evasion.

**FIG 4:**
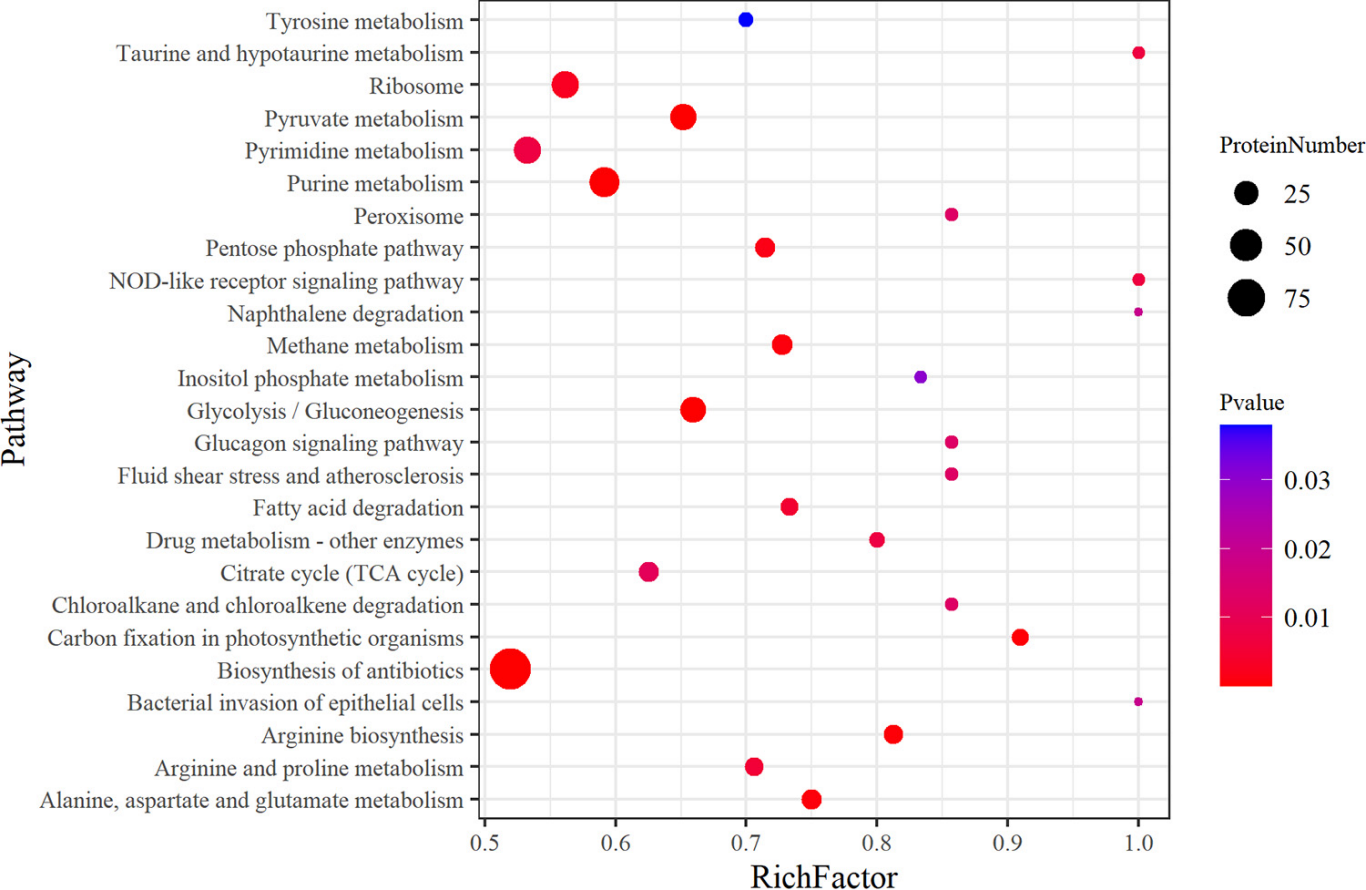
KEGG enrichment analysis of proteome DEPs of S. aureus SCV strain IE2 compared to normal strain IE1. RichFactor is the ratio between the number of DEPs and all proteins with pathway annotations, which represents the proportion of DEPs in this pathway. The sizes of the dots denote the number of DEPs, and colors correspond to the *P* value range.

In addition, all identified proteins were subjected to KEGG pathway analysis, and 153 pathways were found involving in 1,672 proteins, including 627 DEPs (Data Set S4). In addition, 25 statistically significant pathways were filtered (*P* < 0.05), as shown in Fig. 4. Similar to the transcriptome results, the enriched pathways were involved mainly in metabolism, including carbohydrate metabolism (five pathways), amino acid metabolism (five pathways), lipid metabolism (one pathway), nucleotide metabolism (two pathways), and energy metabolism (two pathways).

**FIG 5:**
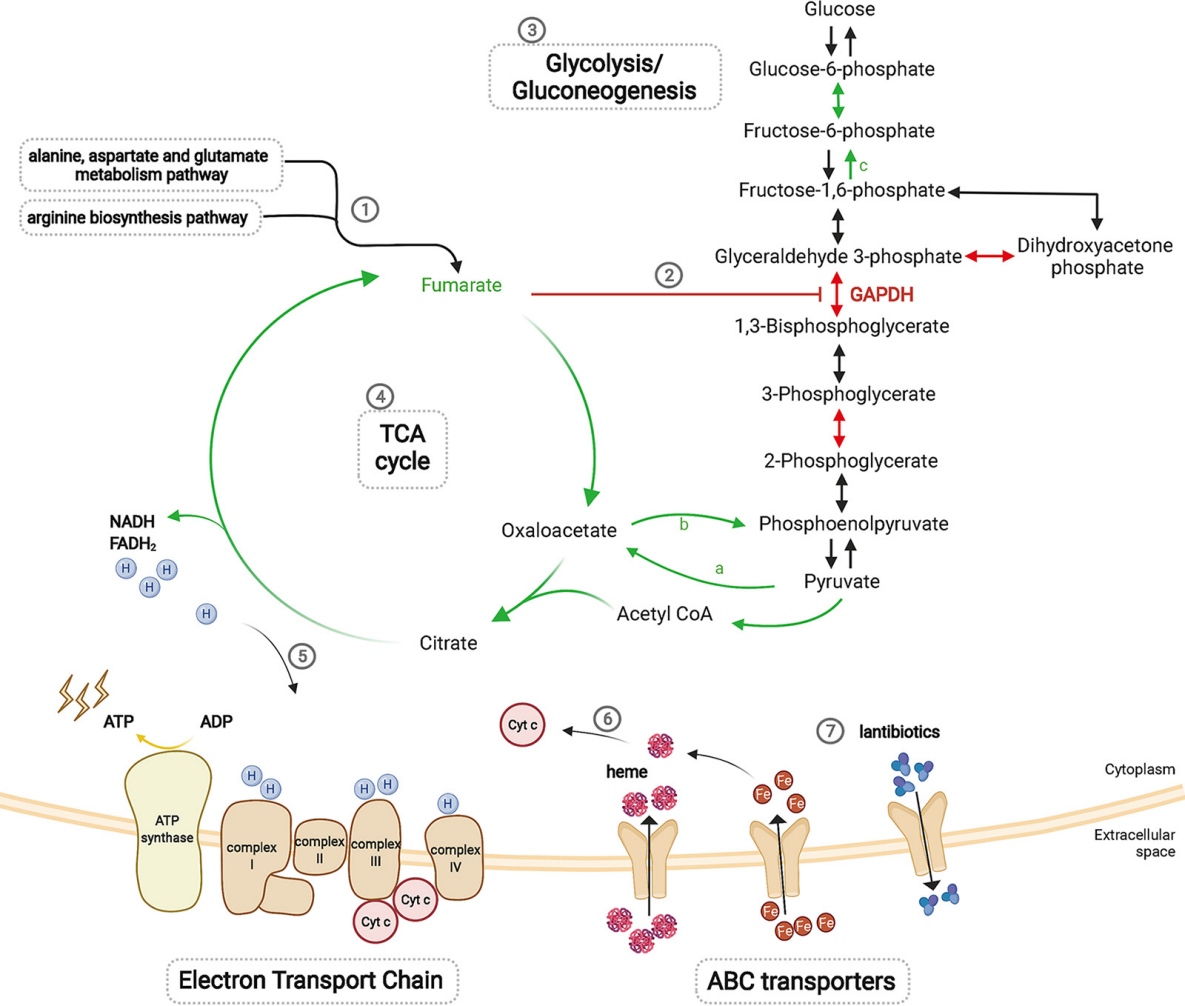
Diagram of metabolic pathways and ABC transporters. (1) Fumarate production was reduced based on the alanine, aspartate, and glutamate metabolism pathway and the arginine biosynthesis pathway. (2) GAPDH was upregulated, and fumarate inhibited glycolysis by inhibiting the glycolysis enzyme GAPDH. (3) Glycolysis was generally upregulated due to the downregulation of most key gluconeogenesis enzymes, including pyruvate carboxylase (a), phosphoenolpyruvate carboxykinase (b), and fructose bisphosphatase (c), in the glycolysis/gluconeogenesis pathway. (4) The TCA cycle was generally downregulated. (5) ATP was reduced in oxidative phosphorylation because the electron transport chain was affected by NADH and FADH_2_ production in the TCA cycle. (6) The electron transport chain was interrupted because downregulated ABC transporters led to iron and heme deficiencies, affecting cytochrome *c*. (7) Downregulated lantibiotic ABC transporters led to the intracellular accumulation of lantibiotics.

For the glycolysis/gluconeogenesis pathway, three-quarters of the key gluconeogenesis enzymes were downregulated, including pyruvate carboxylase, phosphoenolpyruvate carboxykinase, and fructose bisphosphatase (Table S3), while key glycolysis enzymes showed no significant difference, indicating that the glycolysis/gluconeogenesis reaction proceeded more in the direction of glycolysis (Fig. 5; Fig. S4) and that glycolysis was generally upregulated. In addition, it was obvious that the citrate cycle (tricarboxylic acid [TCA] cycle) was downregulated according to KEGG pathway analysis (Fig. 5; Fig. S5).

**FIG 6:**
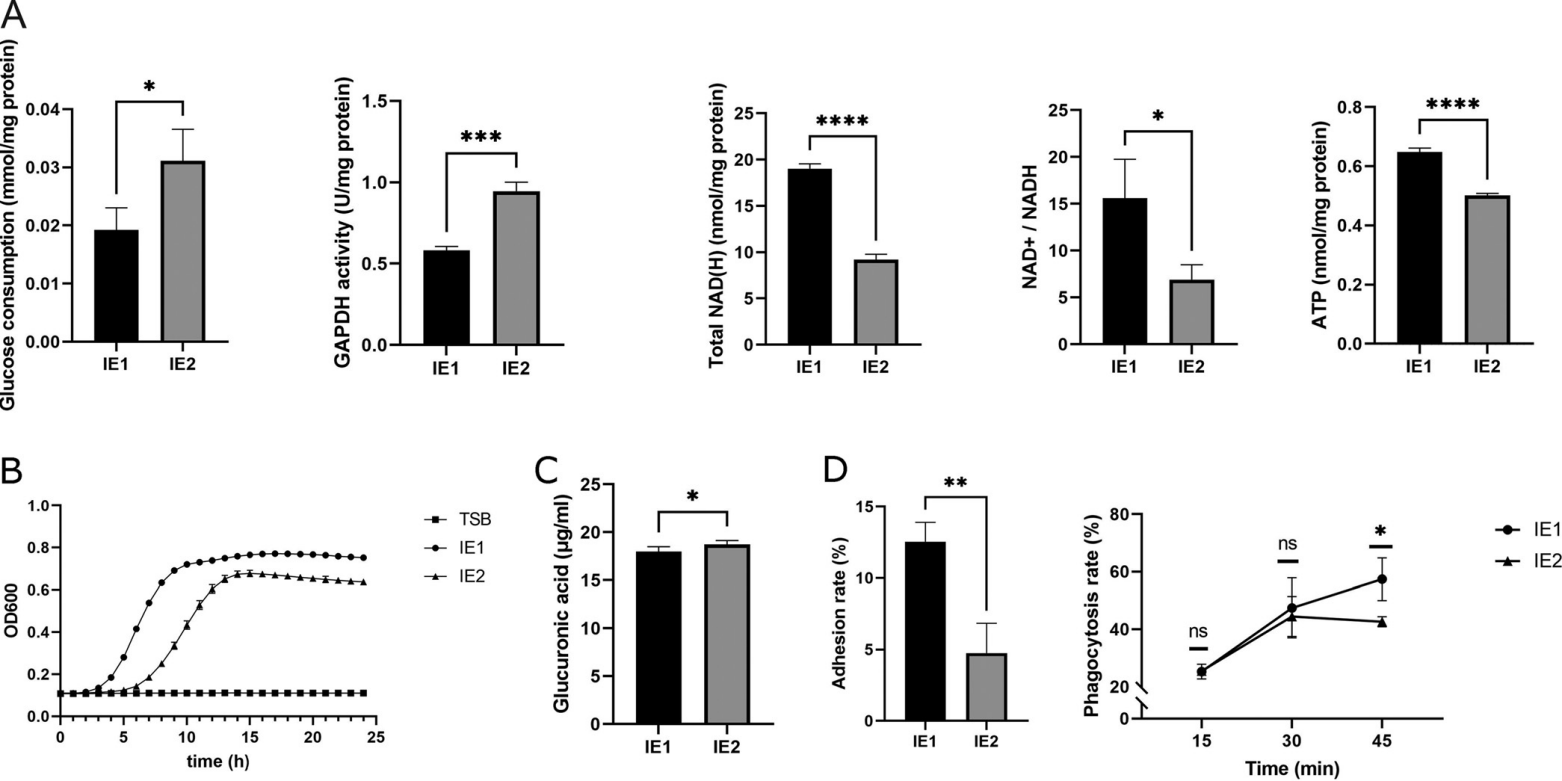
Phenotypic experiments on IE1 and IE2. (A) The metabolism of the two strains changed in glycolysis and the TCA cycle. (B) Growth kinetics were assessed by a growth curve for 24 h. (C) Capsular polysaccharides were determined by glucuronic acid. (D) Adhesion and phagocytosis of S. aureus by host cells. *, *P* < 0.05; **, *P* < 0.01; ***, *P* < 0.001; ****, *P* < 0.0001. ns, not significant.

Metabolic experiments were performed to verify our hypothesis (Fig. 6A). Consistent with the proteome analysis, glyceraldehyde-3-phosphate dehydrogenase (GAPDH) was upregulated in IE2 cells, which was also detected by an enzyme-linked immunosorbent assay (ELISA). Moreover, the increased glucose consumption of IE2 reflects the upregulation of glycolysis. Total NAD(H) was reduced in IE2, and NADH production and utilization were both decreased because of TCA cycle downregulation and electron transport chain deficiencies. NADH was subsequently transformed into NAD^+^, but the deficiency of the electron transport chain led to NAD^+^ downregulation and an NAD^+^/NADH ratio unbalance. Under aerobic conditions, the NAD^+^/NADH ratio is generally lower in pyruvate culture than in glucose culture (15). Due to glycolysis upregulation and TCA cycle downregulation, pyruvate accumulated, resulting in a lower NAD^+^/NADH ratio (Fig. 6A). The production of ATP was significantly downregulated in IE2, indicating reduced metabolism and reflecting the significantly decreased growth rate (Fig. 6B).

### Transcriptome and proteome correlation analysis.

Of all proteins and genes quantified in SCV strain IE2 compared with normal strain IE1 (Table S4), there was a correlation between 2,081 proteins and genes (Data Set S5 and Fig. S6a). Of these, 286 DEGs and DEPs were correlated (Fig. S6b). Correlation analysis of these DEPs and DEGs of IE2 revealed that the expression levels of the proteome and transcriptome showed a good correlation (*R* [Spearman] = 0.6545) (Fig. S7). In fact, there was a complex relationship between RNA and protein expression instead of a linear relationship, which could be divided into five groups (Fig. S8). The expression of 216 genes followed the same trend in both proteome and transcriptome analyses. Of these, 115 genes were downregulated and 101 were upregulated, showing a high level of consistency between protein expression and gene expression (*R* [Spearman] = 0.8533). In addition, 70 genes were expressed in an opposite trend of protein and RNA expression, showing high negative consistency (*R* [Spearman] = −0.6610). Nevertheless, protein and transcriptional expression trends were not consistent for some genes, suggesting the presence of complex regulatory mechanisms in the process of translating mRNA to protein in some genes.

All DEGs and DEPs identified in the transcriptome and proteome of IE2 cells were subjected to cluster analysis (Fig. S9). It was clear that most genes were both up- and downregulated at the transcriptome and proteome levels, although their expression levels were not the same. A heat map revealed that IE2 expressed more downregulated genes than upregulated genes in the transcriptome, while this difference was minimal at the proteome level. Compared to normal strain IE1, the general transcriptome and proteome profiles of SCV strain IE2 were downregulated.

**FIG 7:**
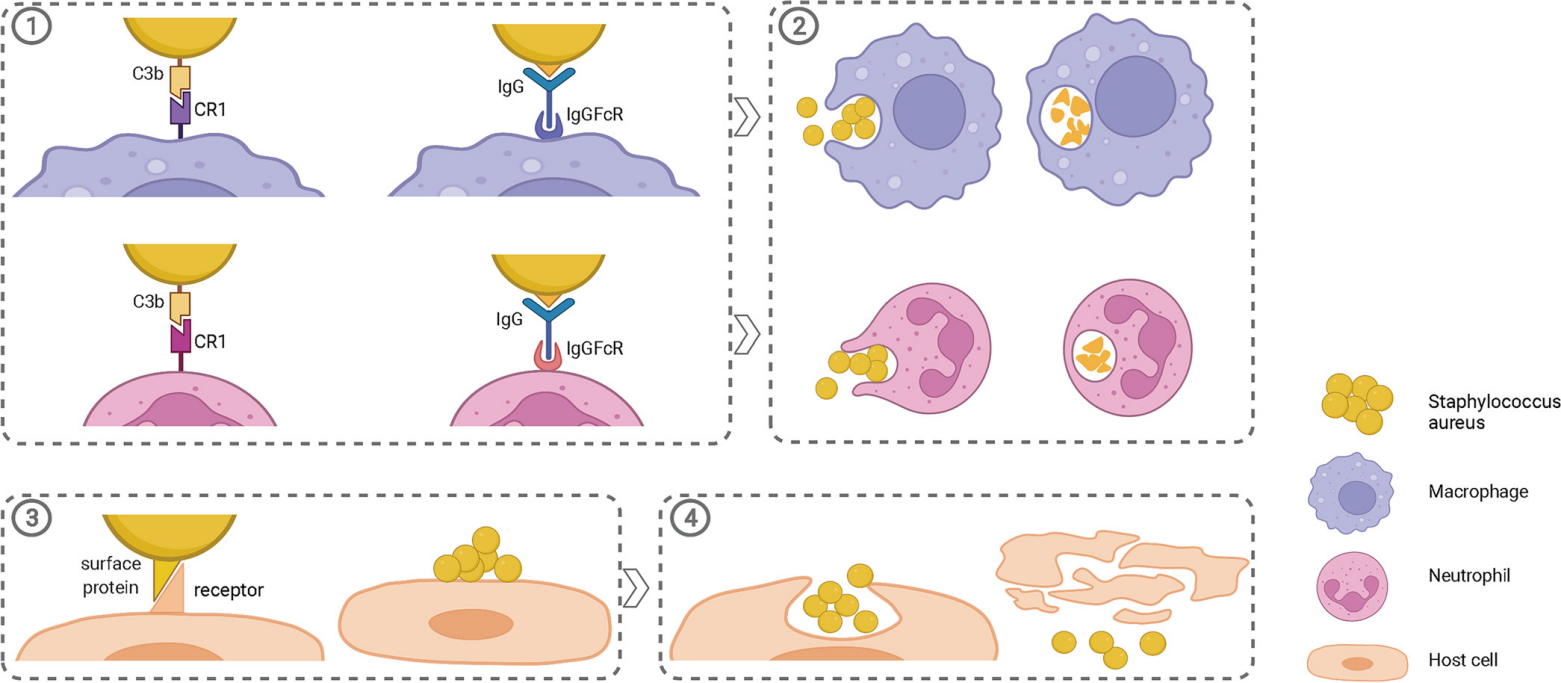
Phagocytosis, adhesion, and invasion between Staphylococcus aureus with phagocytes and nonphagocytic host cells. (1) Opsonization revealed that phagocytic cells phagocytose S. aureus, mediated by antibodies or complement. (2) Phagocytosis of macrophages and neutrophils. (3) Adhesion of S. aureus to host cells through surface proteins. (4) Invasion of host cells by S. aureus. All processes described above were downregulated in SCVs due to capsule polysaccharide upregulation and surface protein downregulation.

We further screened the correlated DEGs and DEPs expressed in the same manner. Setting a gene ratio of ≥ 4 or ≤ −4 and a *P* value of <0.01 as thresholds to filter DEGs and a protein ratio of ≥4 or ≤0.25 and a *Q* value of <0.01 to filter DEPs, a total of seven genes were obtained, with three genes being upregulated and four genes being downregulated at both the transcriptome and proteome levels (Data Set S5). All upregulation was related to capsular polysaccharide biosynthesis, which could prevent phagocytosis. In addition to RT-qPCR, capsular polysaccharide was upregulated in IE2 cells, leading to antiphagocytosis by human neutrophils during coincubation for 45 min (Fig. 6C and D). Moreover, the downregulated DEGs and DEPs were closely related to ABC transporters and surface proteins involved in adhesion to host cells (Fig. 7). Human skin epithelial HaCaT cells adhered less to IE2 than to IE1 (Fig. 6D).

KEGG pathway analysis of the SCV strain IE2 proteome and transcriptome predicted that proteins were involved in 153 pathways, 30 of which were significant (*P* value of <0.05). Genes were involved in 108 pathways, with 24 pathways being found to be statistically significant (*P* value of <0.05). In addition, 100 pathways were related to both the proteome and the transcriptome (Data Set S6), and 7 were statistically significant (*P* value of <0.05 for both). Of these, four were considered the most statistically significant and meaningful pathways, including alanine, aspartate, and glutamate metabolism; antibiotic biosynthesis; arginine biosynthesis; and ribosome biogenesis (Table S5). In the alanine, aspartate, and glutamate metabolism pathway, l-aspartate was converted into adenylosuccinate and l-argininosuccinate, and further conversion into fumarate was inhibited (Fig. S10 and Table S3). In the arginine biosynthesis pathway, citrulline was converted to l-arginosuccinate, which suppressed the production of fumarate (Fig. S11 and Table S3), resulting in the downregulation of fumarate (Fig. 5).

## DISCUSSION

Compared to MRSA ST239 strain IE1, which has a normal colony phenotype, strain IE2 showed a very-small-colony, nonhemolytic phenotype with altered metabolism. As SCV strains have become a major clinical problem, an increasing number of studies are being conducted to elucidate their formation and functional mechanisms. Based on transcriptome or proteome technology, many studies have already identified the altered metabolome of an SCV strain selected with tea tree oil (16), compared the genome and transcriptome of an initial S. aureus WT isolate and stable SCV relapse isolates from a prosthetic joint infection (17), and conducted proteomic analyses with SCV phenotypes (e.g., clinically derived SCVs, *hemB* mutant, and gentamicin-induced SCVs) and normal strains (e.g., clinical wild-type strain, complemented *hemB* mutant, and spontaneous revertant of clinical SCVs) (18). In our study, taking advantage of advanced technologies such as RNA sequencing and protein mass spectrometry, we obtained the transcriptomic and proteomic profiles of normal strain IE1 and SCV strain IE2. Bioinformatics analysis was performed on the transcriptome and proteome, combined with functional assays, to identify key genes that were significantly regulated at both the transcriptional and translational levels in SCV strain IE2, especially genes encoding ABC transporters and capsular polysaccharide (CP), and determine the most enriched pathways closely related to the formation of SCVs, which are involved mainly in metabolic pathways.

Based on our analysis, we propose two hypotheses. First, on the one hand, the inhibition of glycolysis was weakened due to the decrease in fumarate production, and the reaction was directed toward glycolysis in the glycolysis/gluconeogenesis pathway of SCVs, both resulting in glycolysis upregulation. On the other hand, the downregulation of the TCA cycle pathway resulted in reduced NADH and FADH_2_ production, which affected the electron transport chain; due to the downregulation of the ABC transporter, iron and heme production was reduced, which led to the reduction of cytochrome *c* (Cyt *c*) and impaired the electron transport chain. Both processes reduced ATP synthesis, resulting in a dependence on glycolysis to supply energy to SCVs (Fig. 5). Second, the upregulated biosynthesis of capsular polysaccharide inhibited the interaction between complement fragments or antibodies and phagocyte surface receptors, which prevented phagocytosis. Many surface proteins were downregulated and masked by the capsule, which led to reduced adhesion to host cells, facilitating immune evasion by SCVs (Fig. 7).

### ABC transporter downregulation decreased heme and metal levels and weakened self-protection against lantibiotics.

ABC transporters act as transport ATPases with key functions in bacteria and other organisms. On the one hand, they can serve as importers that transport some essential nutrients, such as inorganic and organic ions, sugars, amino acids, peptides, metal ions, and vitamins (19), playing an important role in bacterial metabolism. On the other hand, similar to exporters, they can export many substances out of the cytoplasm, including protein toxins and some drugs (19), which are often responsible for antibiotic resistance (20). The function of most bacteria requires ABC transporters, which are involved in the uptake of siderophores, heme, and vitamin B_12_ (21). Heme is a necessary component of the electron transport chain in bacteria because it is a prosthetic group of cytochromes.

According to our findings, after filtering, we found that some proteins were associated with heme transport or synthesis, such as the haptoglobin-binding heme uptake protein HarA (*harA*) and iron-regulated surface determinant proteins (*isdA* and *isdC*). The iron ABC transporter substrate-binding protein shows a high affinity and a high substrate specificity for iron, which is essential for heme production. Presumably, the dysfunction of heme, which contributes to SCV phenotype formation, has a great deal to do with the downregulation of these proteins and ABC transporters. Moreover, there was a high level of consistency in the significant downregulation of both transcriptome and proteome levels of iron-regulated surface determinant protein A (IsdA) (heme transporter), leading to decreased heme transport. Heme deficiency interrupts the electron transport chain (13), which limits ATP production by oxidative phosphorylation and eventually causes ATP depletion (Fig. 3). This process is related to the persistence of S. aureus (22), and the disabled electron transport system allows S. aureus SCVs to resist aminoglycosides (23).

Metals are common enzymatic cofactors, and their acquisition is essential for organisms to maintain normal metabolism. As mentioned above, iron is closely related to ABC transporters and heme, but not all ABC transporters are related to iron acquisition, with some exhibiting a higher specificity for other metals such as zinc or manganese (21). In addition, a previous study (24) identified novel cobalt and nickel transporters in S. aureus. In our study, a nickel ABC transporter, nickel/metallophore periplasmic binding protein (*cntA*), was downregulated. Nickel is a transition metal required as a cofactor for several bacterial enzymes, and S. aureus expresses the nickel ABC transporter Nik, which functions in metal-replete medium and is necessary for nickel urease activity and urinary tract colonization (24).

In addition, some ABC transporters are related to a special class of peptide antibiotics, lantibiotics, synthesized by ribosomes (20). ABC transporters are involved mainly in the export of lantibiotic precursors and the self-protection of the lantibiotic producer (20). Lantibiotics are generally described as staphylococcins belonging to class I whose precursor peptides undergo posttranslational modifications, which can be quite extensive (25). They may attack various pathogens and are involved in many infectious diseases (25). A previous study (26) found that SCV cells induce the synthesis of lantibiotics. Based on transcriptome analyses, a gene contributing to lantibiotic biosynthesis by encoding lantibiotic dehydratase was slightly upregulated. However, the synthesis and secretion of bacteriocin consume energy, and there is also a risk of damaging the producer by disrupting membrane integrity (25). Thus, there is a high cost for producing cells, and lantibiotic production is beneficial only if required for competitor suppression (27). Therefore, the increase in lantibiotics in SCVs is expected to suppress their rapidly growing competitors to reduce the host immune response (26). Moreover, the decrease in the lantibiotic ABC transporter ATP-binding protein (*ecsA*) at both the transcriptome and proteome levels in SCV strains in our study suggested a weakened immune response against lantibiotics. If the immune system cannot appropriately respond to high lantibiotic concentrations, this may partially inhibit cell growth (25). Thus, we supposed that the upregulation of lantibiotic biosynthesis and the significant downregulation of lantibiotic ABC transporter ATP-binding protein probably caused lantibiotic accumulation and impaired protection for producers, thus influencing the low growth rate of SCVs.

### Glycolysis was upregulated and metabolism was generally downregulated.

According to KEGG pathway analysis, numerous metabolic pathways altered in SCV strain IE2 were prominently involved in amino acid metabolism (Fig. 3 and 4). Fumarate levels decreased according to alanine, aspartate, and glutamate metabolism and arginine biosynthesis pathways. GAPDH, a glycolytic enzyme, was upregulated in the glycolysis/gluconeogenesis pathway (see Fig. S4 in the supplemental material), which could be inactivated by fumarate and downregulate glycolysis (28). Thus, the inhibition of fumarate production upregulated glycolysis. Some previous studies (12, 13, 18) reported that glycolysis was upregulated in S. aureus SCVs, which we verified based on glycolysis/gluconeogenesis pathway analysis (Fig. 5) and functional assays (Fig. 6A). Moreover, it has been reported (29) that Δ*hemB* SCVs can stimulate host cell glycolytic activity and induce necroptosis, which does not kill the germ but releases viable organisms (30), contributing to SCV persistence. The lower levels of fumarate associated with Δ*hemB* infection are the combined products of both host and pathogen metabolisms (29). Therefore, S. aureus SCV formation depends on glycolysis due to electron transport chain deficiencies.

Moreover, as a key link between carbohydrate, amino acid, and lipid metabolism and the final metabolic pathway of the three nutrients, the citrate cycle (TCA cycle) pathway was downregulated in our analysis, and ATP production was decreased significantly, which is similar to the results of other reports (13, 17, 18). Therefore, these results indicated that metabolic activity was generally decreased in the SCV strain.

### Capsular polysaccharide overexpression and surface protein downregulation resist phagocytosis, thereby evading immunity.

Based on the transcriptome, proteome, and correlation results, IE2 significantly upregulated the expression of a CP biosynthesis protein (*cap8*). Cap5 and Cap8 were previously reported to be found in the majority of S. aureus clinical isolates from humans (31, 32), which is consistent with previous research that found that the genes for capsular polysaccharide synthesis were upregulated in the *hemB* mutant compared to the parental strain (12). However, there have been contradictory reports on capsule production when switching to SCVs (26, 33, 34). Diverse factors contributing to capsule production, or more complex mechanisms, may be involved in different strains. Capsular production prevents phagocytosis (35–37). The capsule hinders the interaction between complement C3b and antibodies and their receptors on phagocytes (35, 37, 38). In the complement cascade, C3 is cleaved by C3 convertases to C3b, which then binds to organisms and is recognized by specific receptors on phagocytes. In addition, antibody Fab regions can specifically bind to pathogens, and Fc regions combine with phagocytes, mediating the endocytosis of bacteria by phagocytes. Complement and antibodies are important serum opsonins that facilitate the opsonophagocytic process. Moreover, S. aureus capsules can promote abscess formation (39). Thus, the S. aureus capsule can escape the immune system by impeding phagocytosis (Fig. 6D and Fig. 7), contributing to bacterial persistence in the bloodstream of infected hosts (38), which is of vital importance for the pathogenesis of S. aureus infections.

Furthermore, S. aureus can express various surface proteins, also called cell wall-anchored (CWA) proteins, because they are covalently attached to peptidoglycans (40). The expression of CWA proteins can be altered by growth conditions (40) such as iron-limited conditions and the exponential or stationary phase of growth. These surface proteins contribute to the adhesion to and invasion of host cells (40); therefore, they are known as adhesins. In our study, the ability of IE2 to adhere to HaCaT cells was decreased, and some genes or proteins related to adhesion were significantly downregulated after further filtering, such as the cell wall surface anchor family protein with the LPXTG cell wall anchor motif (*sasD*). Moreover, the iron-regulated surface determinant protein (*isdA*) mediates the adhesion of S. aureus and is involved in heme transport (40). Adherence is also correlated with phagocytosis; thus, these adhesins are unnecessary or even detrimental for the bacteria because they can facilitate attachment to phagocytic cells (41). Therefore, the downregulation of these genes and proteins is beneficial for the survival of SCVs. Moreover, the upregulation of capsules masks surface proteins to prevent adhesion. In addition, a previous study (42) concluded that staphylococcal capsules were capable of inhibiting cell invasion mainly by shielding surface proteins such as fibronectin-binding proteins, which play an important role in invasion by S. aureus. The levels of cell invasion in different cell lines followed a trend similar to that of adhesion, indicating a link between adhesion and invasion events (43). Therefore, the upregulation of capsular polysaccharides and the downregulation of surface proteins may play important roles in reducing adhesion and subsequent invasion during infection of suitable host cells (Fig. 7). However, it is worth noting that the adhesion capacity of S. aureus does not always correlate well with its invasion capability (44). Further research is required to confirm these findings.

Above all, changes in ABC transporters, capsular polysaccharide biosynthesis, and metabolism play important roles in the formation and persistence of SCVs. The electron transport chain was interrupted, probably because the downregulation of the ABC transporter affected iron and heme transport, which in turn reduced heme synthesis. Heme is the prosthetic group of cytochromes, which is part of the electron transport chain. Furthermore, defects in the electron transport chain limit oxidative phosphorylation to produce ATP, resulting in glycolysis being dependent on the supply of energy, which could be proven by the upregulation of glycolysis and the downregulation of the inhibitor fumarate. Moreover, the downregulation of the TCA cycle pathway reflected the slower metabolism of the SCV strain. In addition, capsular polysaccharides prevented complement deposition on or antibody binding to the bacterial surface and interactions with their receptors on phagocytes for antiphagocytosis. Conversely, surface proteins that function as adhesins contribute to the adhesion to and invasion of host cells and could be blocked by capsules, which impedes their attachment to phagocytic cells. Thus, the upregulation of capsule polysaccharides and the downregulation of surface proteins contribute to immune evasion, resulting in SCV persistence.

Benefiting from the development of advanced technologies, we have obtained a comprehensive profile of S. aureus SCVs compared to normal strains. However, transcriptomic and proteomic analyses do not correspond to each other, and this inconsistency may reflect limitations of transcriptome and proteomic technologies, such as low sensitivity, missing data, and background noise. In addition, divergent efficiencies at the transcriptional and translational levels may have caused posttranslational turnover and modification (45). Thus, we performed a correlation analysis on the proteome and transcriptome, which could be used for verification to reduce mistakes caused by a single omics analyses, and the results were verified by additional functional assays. Nevertheless, some genes were expressed in an opposite trend of protein and RNA levels, which indicated there were complex regulatory mechanisms involved in the process of translation. This analysis was based on a specific pair of S. aureus strains isolated from a patient, which may be a limitation of our study. Considering that SCVs may exhibit distinct changes based on different disease stages and host environments, our study focused on only one condition, and we did not perform further research on how they may change under different environments.

In conclusion, omics analyses can reveal critical information about bacterial strains, and the application of a dual-omics strategy in this study is expected to provide a basis for more comprehensive research. We identified several key genes and metabolic pathways closely related to SCVs, which are vital for understanding the mechanisms underlying SCV formation and survival. However, the exact regulatory mechanisms of these genes and their possible pathways require further research.

## MATERIALS AND METHODS

This study followed the principles of the Declaration of Helsinki and was approved by the Ethical Committee of Peking University People’s Hospital (2022PHB073). In “flow cytometry assay of phagocytosis by human neutrophils”, we isolated neutrophils from the blood of healthy volunteers.

### Bacterial strains.

MRSA ST239 strain IE1 with a normal colony phenotype and MRSA ST239 strain IE2 with SCVs were isolated from the blood culture of an elderly female patient with subacute bacterial endocarditis. The acquisition of these two strains was described in our previous publication (46), and the patient’s presentation over the disease course is presented in a timeline illustrated in Fig. 1.

### Total RNA extraction.

Purified IE1 and IE2 single colonies were inoculated into tryptic soy broth (TSB) (35°C for 5 h) to reach the exponential growth phase (optical density at 600 nm [OD_600_] = 0.4 to 0.6). Next, 200 μL of the bacterial pellet was collected by centrifugation, resuspended in 50 U/mL lysostaphin (100 μL), and incubated (37°C) for 30 min to lyse S. aureus cell walls. Total RNA was extracted using an RNeasy minikit (Qiagen, Hilden, Germany), and rRNA was removed using a Ribo-Zero magnetic kit (Zhongbei Linge Technology Development Co. Ltd., Beijing, China). RNA integrity was determined using agarose gel electrophoresis (AGE). The RNA purity and concentration were determined using a NanoDrop2000 spectrophotometer (Thermo Fisher Scientific, USA).

### RNA sequencing and bioinformatics analysis.

The RNA library was constructed using the TruSeq RNA sample preparation kit (Illumina, San Diego, CA, USA) and sequenced on a Hiseq4000 platform (Illumina, San Diego, CA). The filtered clean data were mapped to the TW20 reference genome (GenBank accession number FN433596) using HISAT (47). Fragments per kilobase of exon model per million mapped reads were calculated for each gene to assess gene expression levels. *P*_adj_ refers to the false discovery rate (FDR)-adjusted *P* value; *P*_adj_ values of ≤0.05 and fold changes of ≥2.0 were set as thresholds to identify differentially expressed genes (DEGs). Cluster analysis of DEGs was performed to identify genes with similar functions. The Spearman algorithm was used to compare samples, and the Pearson algorithm was used to compare genes. The DEGs were then subjected to Kyoto Encyclopedia of Genes and Genomes (KEGG) enrichment analysis to determine their involvement in metabolic pathways. KOBAS tools were used for KEGG pathway enrichment analysis. Fisher’s exact test was performed, and a *P* value of *≤*0.05 was defined as a threshold to filter statistically significant enrichment pathways.

### Protein mass spectrometry and bioinformatics analysis.

IE1 and IE2 proteins were extracted using ice bath ultrasound (48, 49). Proteins were digested by trypsin into peptides labeled with iTRAQ reagent (Applied Biosystems, Foster City, CA, USA); next, the labeled peptides were fractionated using the LC-20AB unit (Shimadzu Company, Kyoto, Japan) and detected using the TripleTOF 5600 electrospray ionization-tandem mass spectrometry system (Sciex, Framingham, MA, USA). Proteins were identified by Mascot 2.3.02 (Matrix Science, London, UK) using a protein sequence database and were quantified by iTRAQ using IQuant software (50). Experiments were repeated three times. Significant spectra and peptides were identified by setting a peptide-spectrum match (PSM)-level FDR of ≤0.01, and the false-positivity rate of proteins was controlled by setting a protein-level FDR of ≤0.01 using the picked-protein FDR approach (51).

Differentially expressed proteins (DEPs) were filtered by a fold change of >1.2 and a *Q* value of <0.05 in at least two of three repeated experiments. The enriched KEGG pathways of DEPs were determined using a hypergeometric test with a threshold *P* value of <0.05.

### Proteome and transcriptome correlation analysis.

Spearman’s rank correlation coefficient (52) between gene and protein expression was calculated, and cluster analysis was performed on proteins and genes. Moreover, the significantly enriched pathways in the KEGG database were annotated and identified using a *P* value of <0.05. The main parameters for the correlation analysis are listed in Table S6 in the supplemental material.

### Whole-genome sequencing (WGS).

The total genomic DNA of the IE1 and IE2 strains was extracted using a TIANamp bacterial DNA kit (Tiangen Biotech Co. Ltd., Beijing, China). The two strains were sequenced using Illumina technology with a 150-bp paired-end protocol on an Illumina NextSeq instrument (Illumina Inc., San Diego, CA, USA). The Mash tool (53) was used to identify the best-matching chromosomal reference. The reads were mapped, and single nucleotide polymorphisms (SNPs) were identified. The Genome Analysis Toolkit (GATK) (54) was used to filter low-quality data. All SNPs identified via bioinformatics analysis were verified using the Integrative Genomics Viewer (IGV) (55) and confirmed by PCR and Sanger sequencing.

Moreover, the contigs of the genome were annotated using Prokka (56), and the core genome was identified using Roary (57). Alignments were screened for recombination using ClonalFrameML (58), and putative recombinant regions were removed before further phylogenetic analyses. Maximum likelihood phylogenetic trees were constructed using RAxML (59).

### Growth assay.

S. aureus strains IE1 and IE2 were cultured in TSB to the exponential growth phase (OD_600_ = 0.4 to 0.6). Cultures were diluted 1:10,000 until the OD_600_ was close to that of the TSB blank control and were grown at 37°C. The cell density was determined every 30 min for 24 h by measuring the OD_600_.

### RT-qPCR.

After the extraction of total RNA, cDNA was generated using PrimeScript RT master mix (TaKaRa). qPCR was performed in triplicate using TB green premix Ex *Taq* II (TaKaRa) on a 7500/7500 Fast real-time PCR system (Applied Biosystems, CA, USA) according to the manufacturer’s instructions. Relative gene expression was calculated from standard curves and the comparative threshold cycle (*C_T_*) method, and the *gyrB* gene was used as an internal control for normalization.

### Intracellular ATP and NAD^+^/NADH concentrations.

The IE1 and IE2 strains were cultured in TSB to the exponential growth phase, precipitated, and resuspended in phosphate-buffered saline (PBS). ATP and NAD^+^/NADH concentrations were measured in triplicate using the ATP assay kit (Beyotime) and the NAD^+^/NADH assay kit with WST-8 (Beyotime), respectively, according to the manufacturer’s instructions. The total protein content in each sample was measured using the enhanced bicinchoninic acid (BCA) protein assay kit (Beyotime) for normalization.

### Glucose consumption.

The method for determining glucose consumption was conducted as previously described (60), with some modifications. IE1 and IE2 were cultured overnight, diluted in fresh TSB (1:50), and placed into a shaking incubator at 37°C. Considering their growth kinetics, 1-mL cultures were removed at different times when the OD_600_ was the same, as determined by continuous monitoring. The medium supernatants were then used to measure the glucose concentration in triplicate using an AU5800 chemistry analyzer (Beckman Coulter, Brea, CA, USA). Every time the medium was removed, proteins were extracted from the bacterial pellets and measured using an enhanced BCA protein assay kit (Beyotime). Next, the glucose consumption required to produce the same amount of total bacterial protein was calculated.

### GAPDH determination.

The active proteins of IE1 and IE2 were extracted using the one-step bacterial active protein extraction kit (Sangon Biotech). The glyceraldehyde-3-phosphate dehydrogenase (GAPDH) concentration was measured in triplicate by an ELISA using a bacterial GAPDH ELISA kit (Shanghai Fantai Biotechnology Co. Ltd.) according to the manufacturer’s instructions. Next, the total protein concentration in each sample was normalized using the enhanced BCA protein assay kit (Beyotime).

### Capsular polysaccharide extraction and quantification.

The method for the extraction and quantification of capsular polysaccharide was carried out as previously described (61), with some modifications. Bacteria in the late growth phase were collected and washed with PBS, followed by autoclaving at 121°C for 60 min and centrifugation. The supernatant was collected and filtered through a 0.2-μm-pore-size membrane. Next, CTAB (cetyl trimethyl ammonium bromide) (1%) was added to the polysaccharides. The sediment was collected by centrifugation and added to 1 mol/L CaCl_2_ to dissociate the polysaccharides from CTAB, followed by centrifugation. Ethanol was added to the supernatant to a final concentration of 25%, and the mixture was allowed to stand overnight at 4°C. The nucleic acid precipitate was removed by centrifugation. Cooled ethanol was mixed with the supernatant to 80% (vol/vol). The polysaccharide was then precipitated and washed with absolute ethanol and acetone.

Capsular polysaccharide was dissolved in water and quantified using glucuronic acid. Borax (sodium tetraborate) (12.5 mM) in H_2_SO_4_ and a standard glucuronic acid solution were added to the samples. The mixture was vigorously vortexed, boiled for 5 min, and placed on ice for 10 min. Subsequently, 0.15% 3-hydroxydiphenol was added to the mixture. After incubation for 5 min at room temperature, the absorbance was measured at 520 nm.

### Flow cytometry assay of phagocytosis by human neutrophils.

The phagocytosis assay was performed as previously described (62), with some modifications. Cultures grown overnight were inactivated at 70°C for 1 h and washed with PBS. The pelleted bacteria were labeled with 0.1 mg/mL fluorescein isothiocyanate (FITC), which was dissolved in 0.1 M NaHCO_3_ (pH 9.0) at 25°C for 1 h. Free FITC was washed off with PBS. The labeled bacteria were stored at −80°C prior to the experiment.

Peripheral blood polymorphonuclear leukocytes were isolated from the blood of healthy volunteers. In brief, EDTA anticoagulant-treated blood was fractionated by a Ficoll/Hypaque gradient (GE Healthcare) to separate mononuclear cells from neutrophils. Neutrophils were further purified by dextran sedimentation of the red blood cell (RBC) layer before lysis of residual RBCs with lysing buffer (BD). Next, neutrophils were counted and resuspended in PBS.

Phagocytosis was performed using 1.5-mL Eppendorf tubes. Neutrophils were mixed with PBS and healthy human serum in a shaking incubator at 37°C for 10 min. Subsequently, the cells and FITC-labeled bacteria were mixed at a ratio of 1:10 (multiplicity of infection [MOI] = 10) in a final volume of 200 μL. The reaction mixture was incubated at 37°C with shaking for 15 to 45 min. The samples were pelleted and washed with PBS in an ice bath to remove free fluorescent bacteria, and trypan blue (final concentration, 0.004%) was added to quench extracellular fluorescence. The experiments were performed in triplicate.

The samples were analyzed using a FACSCanto cytometer (BD Biosciences, San Jose, CA, USA). Neutrophils were gated by adjusting forward-scatter (FSC) and side-scatter (SSC) parameters. A total of 10,000 events were collected for each sample gated on neutrophils. All data were exported as flow cytometry standard format 3.0 files (FCS files), which were analyzed using FlowJo version 10.6.2 (TreeStar, Ashland, OR, USA). Neutrophils were distributed into two clusters based on their fluorescence intensities.

### Adhesion by HaCaT human skin epithelial cells.

The cell adhesion assay was performed as previously described (63). HaCaT human skin epithelial cells were cultured in high-glucose Dulbecco’s modified Eagle’s medium (DMEM) with fetal bovine serum (10%) at 37°C with 5% CO_2_. Bacteria were collected during the exponential growth phase. Bacterial pellets were suspended in DMEM and mixed with cells at an MOI of 10 in 24-well plates, followed by incubation for 2 h. The supernatants were discarded, and the cells were washed with sterile PBS to remove nonadherent bacteria. Subsequently, the cells were lysed using 0.01% Triton X-100, and the bacterial CFU were enumerated by plating onto tryptic soy agar (TSA) plates. The experiments were performed in triplicate.

### Data availability.

The raw data from WGS and RNA sequencing are available at the NCBI under BioProject accession number PRJNA898597.
