# Supplementary material for: Transcriptome and Proteome of Methicillin-Resistant Staphylococcus aureus Small-Colony Variants Reveal Changed Metabolism and Increased Immune Evasion
Source: Microbiol Spectr. 2023 Feb 14;11(2):e01898-22. doi: 10.1128/spectrum.01898-22 (PMC10101100; doi:10.1128/spectrum.01898-22)
Supplement: Supplemental file 7 — Supplemental material. Download spectrum.01898-22-s0007.pdf, PDF file, 1.2 MB [file spectrum.01898-22-s0007.pdf]

Supplementary Data

Table S1 Single nucleotide polymorphisms (SNPs) of IE2 compared with IE1 according to whole genome bioinformatics analysis.

Gene	Product	Nucleotide mutation	Amino acid variation
prkC	Serine/threonine-protein kinase PrkC	T518C, G519A	V173A
plsY	Glycerol-3-phosphate acyltransferase	T557A	I186N
deoC	2-Deoxyribose-5-phosphate aldolase	G33A, T72G, G82A, T138C	E28K
eap	Extracellular adherence protein	T1134C	None
sstD	Iron compound ABC uptake transporter substrate-binding protein	A947G	D316G
recU	RecU Holliday junction resolvase	C618T	D206=
uvrB	Excinuclease ABC subunit B;	A405G	P135=
hsdM	Type I restriction–modification system, M subunit	T408G, T567C, A572G	H191R
Unknown	Smooth muscle caldesmon	C65T	T22I
spoVG	Putative septation protein spoVG	236_237insATATGATGAAACAGATGAAGTAGTACCAGATAAAAACGCTACATCAGAAGATTCAGA	V79_E80insYDETDEVVPDKNATSEDSE
xerC	Tyrosine recombinase xerC	T797C	V264A
ald	Alanine dehydrogenase	1006_1007insTT	N38Kfs*22
hepT	Heptaprenyl diphosphate synthase component II	340_373del	L114Kfs*8
glmM	Phosphoglucosamine mutase	687del	V230*

Table S2 SNPs of IE2 compared with IE1 that verified using IGV and confirmed by PCR and Sanger sequencing.

Gene	Product	Nucleotide mutation	Amino acid variation
prkC	Serine/threonine-protein kinase PrkC	T518C	V173A
sstD	Iron compound ABC uptake transporter substrate-binding protein	A947G	D316G
recU	RecU Holliday junction resolvase	C618T	D206=
uvrB	Excinuclease ABC subunit B;	A405G	P135=
spoVG	Putative septation protein spoVG	236_237insATATGATGAAACAGATGAAGTAGTACCAGATAAAAACGCTACATCAGAAGATTCAGA	V79_E80insYDETDEVVPDKNATSEDSE
ald	Alanine dehydrogenase	1006_1007insTT	N38Kfs*22
hepT	Heptaprenyl diphosphate synthase component II	340_373del	L114Kfs*8
glmM	Phosphoglucosamine mutase	687del	V230*

Table S3 The major genes and proteins for major pathways.

Genes	Proteins	Up/Down	Padj	Pathways
gapA	Glyceraldehyde 3-phosphate dehydrogenase [EC: 1.2.1.12]	Up	1.61E-10	Glycolysis / gluconeogenesis
pckA	Phosphoenolpyruvate carboxykinase [EC: 4.1.1.49]	Down	1.56E-27	Glycolysis / gluconeogenesis
fbp	Fructose bisphosphatase [EC: 3.1.3.11]	Down	0.007952	Glycolysis / gluconeogenesis
pyc	Pyruvate carboxylase [EC: 6.4.1.1]	Down	3.59E-19	Citrate cycle (TCA cycle)
purA	Adenylosuccinate synthase [EC: 6.3.4.4]	Down	2.96E-21	Alanine, aspartate and glutamate metabolism
argG	Argininosuccinate synthase [EC: 6.3.4.5]	Down	9.48E-17	Alanine, aspartate and glutamate metabolism; Arginine biosynthesis
purB	Adenylosuccinate lyase [EC: 4.3.2.2]	Down	1.64E-19	Alanine, aspartate and glutamate metabolism
argH	Argininosuccinate lyase [EC: 4.3.2.1]	Down	1.64E-10	Alanine, aspartate and glutamate metabolism; Arginine biosynthesis

Table S4 The number of correlated proteins and genes between proteome and transcriptome of S. aureus SCVs strain IE2 compared to wild strain IE1

Groups	Type	Number of Proteins	Number of Genes	Number of Correlations
IE2 vs IE1	Identification	2163	2697	2081
IE2 vs IE1	Quantitation	2163	2697	2081
IE2 vs IE1	Differentially expressed	773	822	286

Table S5 The most significant KEGG pathways filtered in correlation analysis of proteome and transcriptome of S. aureus SCVs strain IE2 compared to wild strain IE1

Pathway name	Protein P-value	Gene P-value	Proteins number	Genes number	Correlations number
Alanine, aspartate and glutamate metabolism	0.00068516	0.003900094	15	15	13
Biosynthesis of antibiotics	2.00E-05	0.007064807	94	84	50
Arginine biosynthesis	0.000425979	0.028931458	13	12	11
Ribosome	0.002782726	0.041736185	32	28	12

Table S6 The main parameters of correlation analysis

Type	Value
Unique peptide of proteins	1
Fold change of proteins	1.2
P-value of proteins	<0.05
Fold change of genes	2
P-value of genes	<0.05
P-value of pathway enrichment	<0.05
Blast identity	100
Blast E-value	1e-8

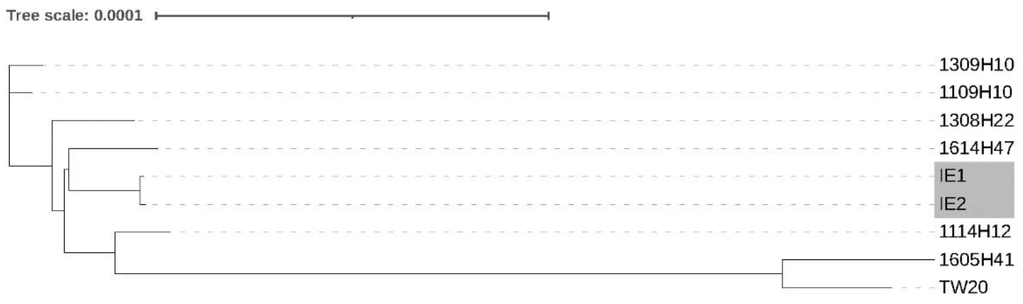

Fig. S1 The phylogenetic trees of IE1 and IE2 with other ST239 strains selected randomly. The strains were clustered on the same branch and were highlighted in grey.

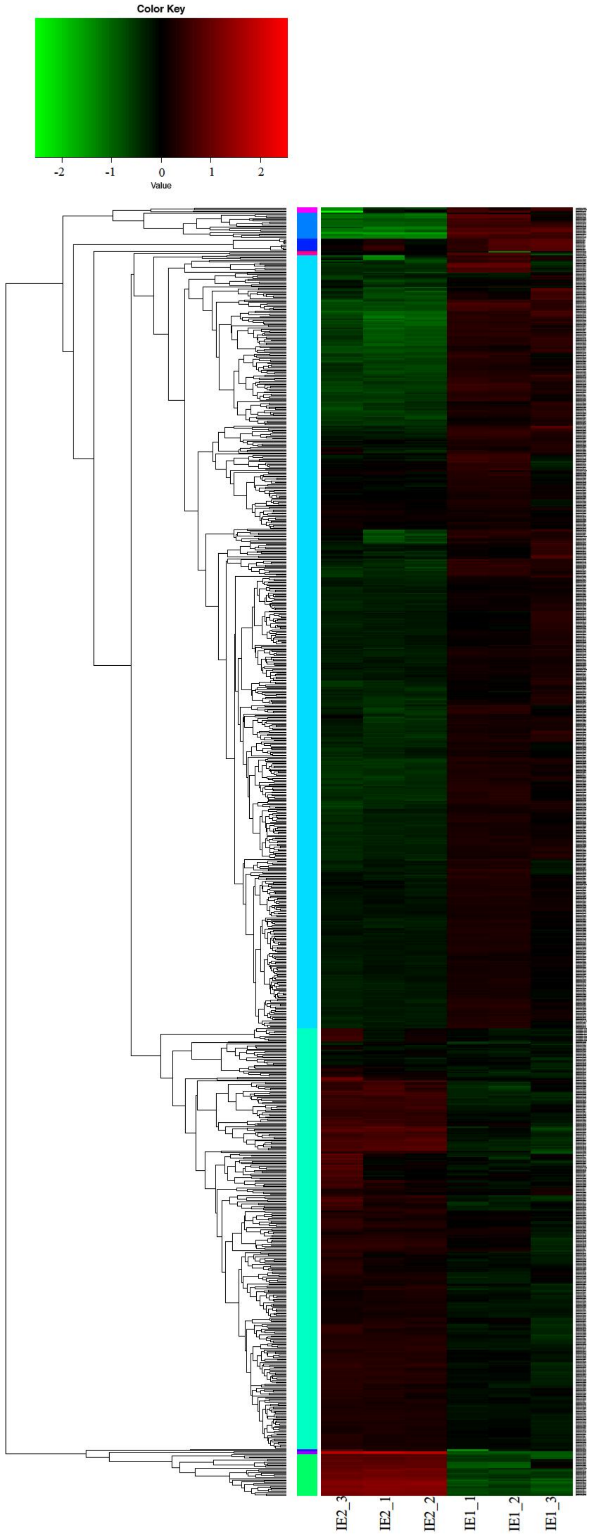

Fig. S2 Cluster analysis of differentially expressed genes (DEGs) in the transcriptome of Staphylococcus aureus wild strain IE1 and SCVs strain IE2. Heatmap colors indicates relative expression (log10 FPKM); the first 3 columns are the repeats of SCVs strain IE2 and the last 3 columns are the repeats of wild strain IE1; the left side is a dendrogram according to expression of transcriptome genes.

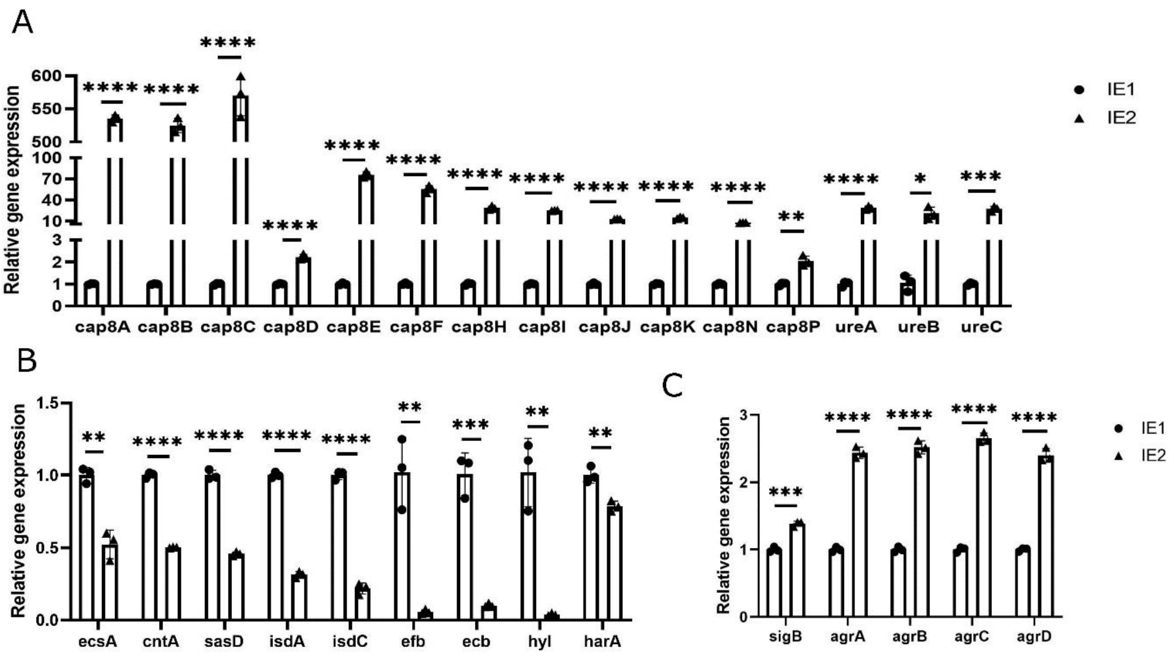

Fig. S3 Relative expression of transcriptome genes in *S. aureus* IE1 and IE2 was measured with qPCR and normalized to gyrB expression. Capsular genes (*cap*) and urease genes (*ure*) were upregulated (A). ABC transporters (*ecsA, cntA*), surface anchored protein (*sasD, isdA* and *isdC*), fibrinogen-binding protein (*efb, ecb*), alpha-hemolysin (*hyl*) and heme uptake protein (*harA*) were downregulated (B). The regulators of capsular were upregulated slightly (C).

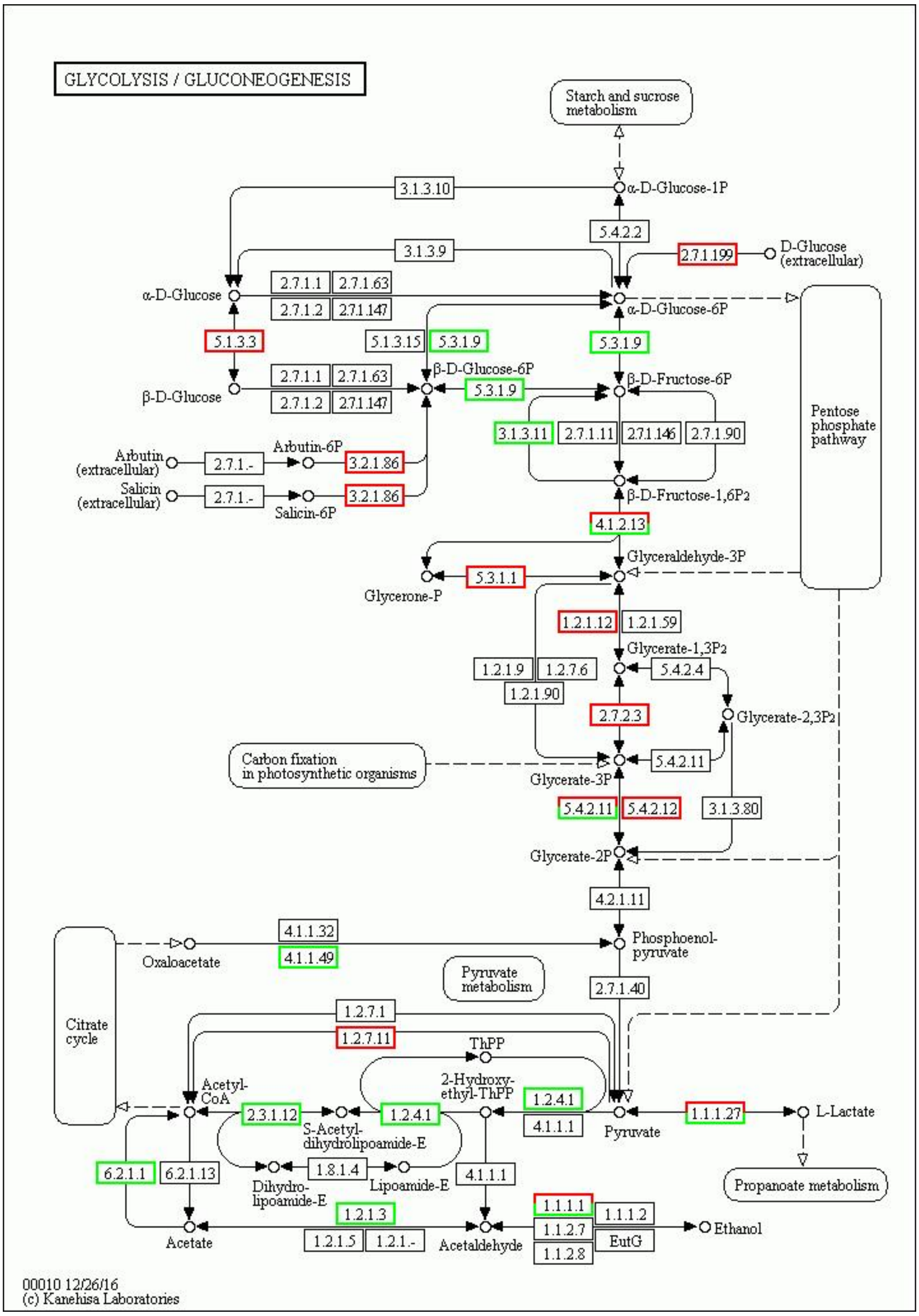

Fig. S4 Glycolysis / gluconeogenesis pathway of S. aureus SCVs proteome from KEGG website. The red boxes indicate that the enzyme is upregulated, and green boxes indicate downregulation. Three quarters of gluconeogenesis key enzymes were down-regulated, including pyruvate carboxylase (6.4.1.1, shown in Fig. S5), phosphoenolpyruvate carboxykinase (4.1.1.49) and fructose bisphosphatase (3.1.3.11), while glycolysis key enzymes showed no significant difference. Glyceraldehyde 3-phosphate dehydrogenase (GAPDH) (1.2.1.12) is upregulated.

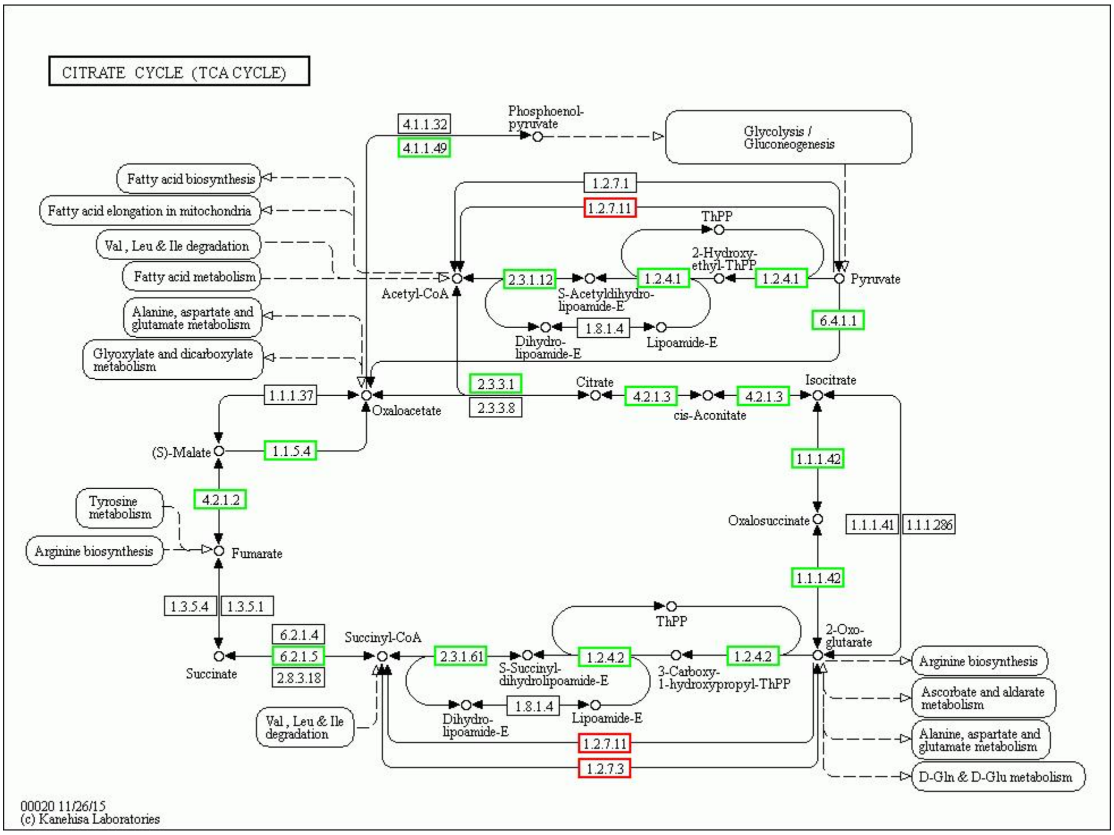

Fig. S5 Citrate cycle (TCA cycle) pathway of S. aureus SCVs proteome from KEGG website. The red boxes indicate that the enzyme is upregulated, and green boxes indicate downregulation. TCA cycle was generally downregulated.

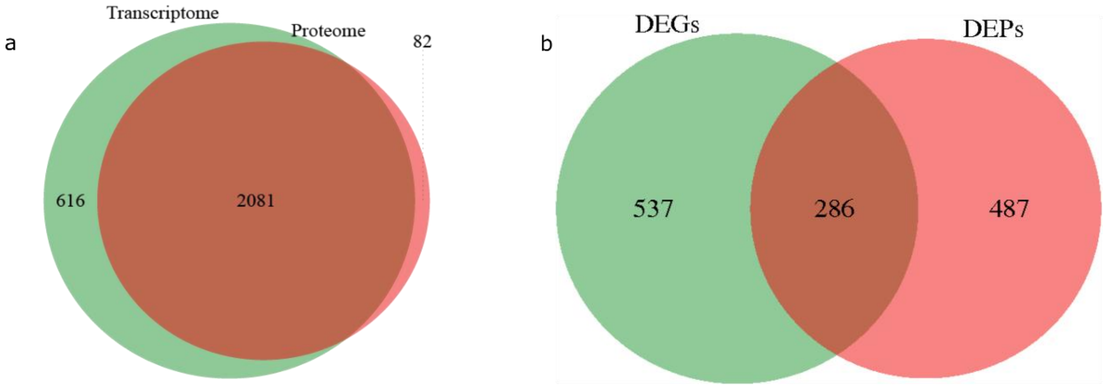

Fig. S6 Venn diagram showing all identified genes and proteins (a) and DEGs and DEPs (b) in transcriptome and proteome levels of MRSA ST239 SCVs strain IE2. DEGs, differentially expressed genes; DEPs, differentially expressed proteins.

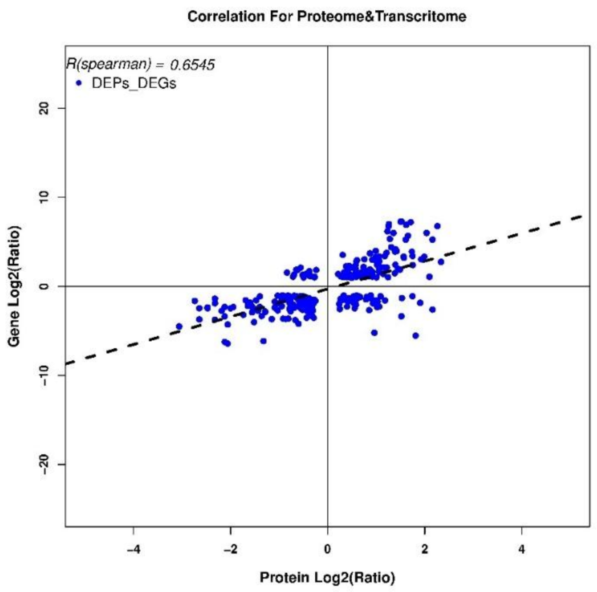

Fig. S7 Correlation between DEPs and DEGs of MRSA ST239 SCVs strain IE2. DEPs, differentially expressed proteins; DEGs, differentially expressed genes.

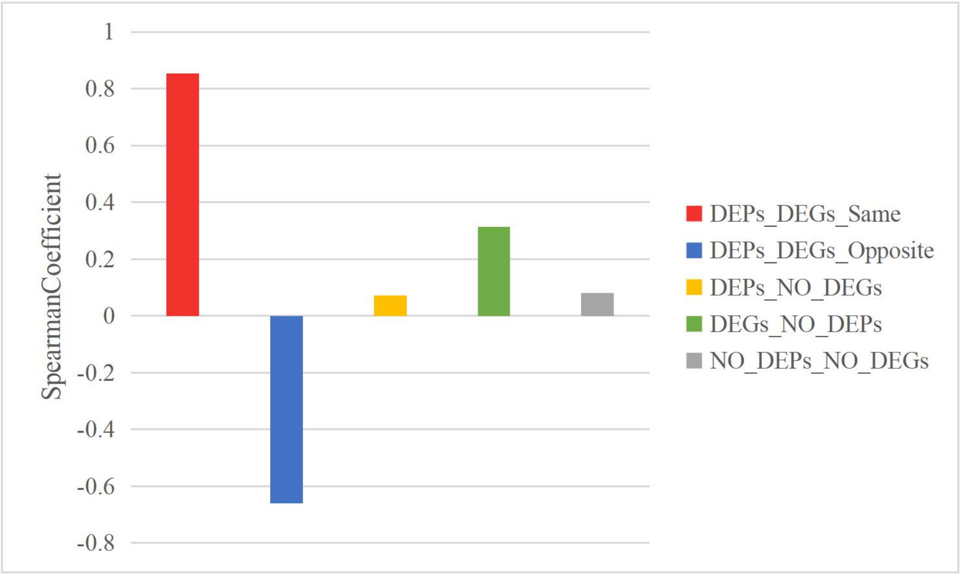

Fig. S8 Five groups can be divided according to proteome and transcriptome correlation analysis. Y-axis means Spearman coefficient; red column represents same trend of protein and RNA expression and has significant differences; blue column represents opposite trend of protein and RNA expression and has significant differences; yellow column represents significant differences in protein expression but no significant differences in RNA expression; green column represents significant differences in RNA expression but no significant differences in protein expression; grey column represents no significant differences in both protein and RNA expression.

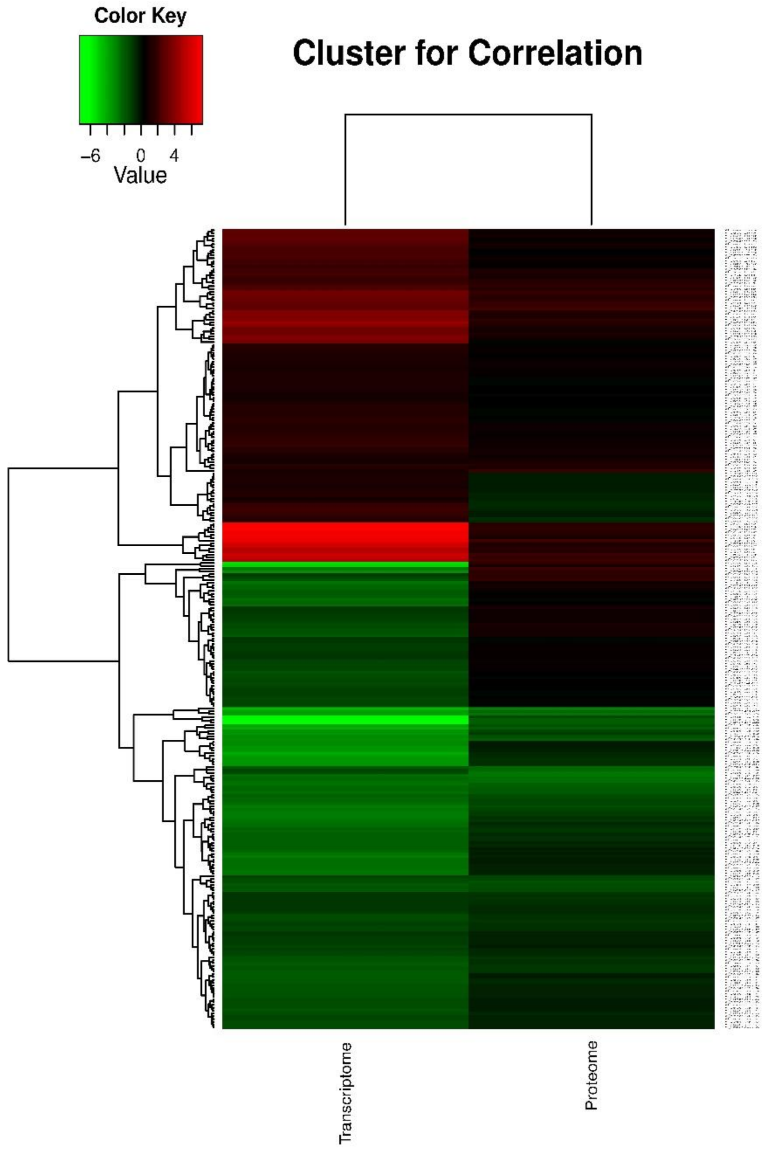

Fig. S9 Heatmap shows cluster analysis of differentially expressed genes (DEGs) and differentially expressed proteins (DEPs) of MRSA ST239 SCVs strain IE2. Colors indicate relative abundance of RNA or proteins; On the left is a clustered dendrogram according to the expression of transcriptome and proteome.

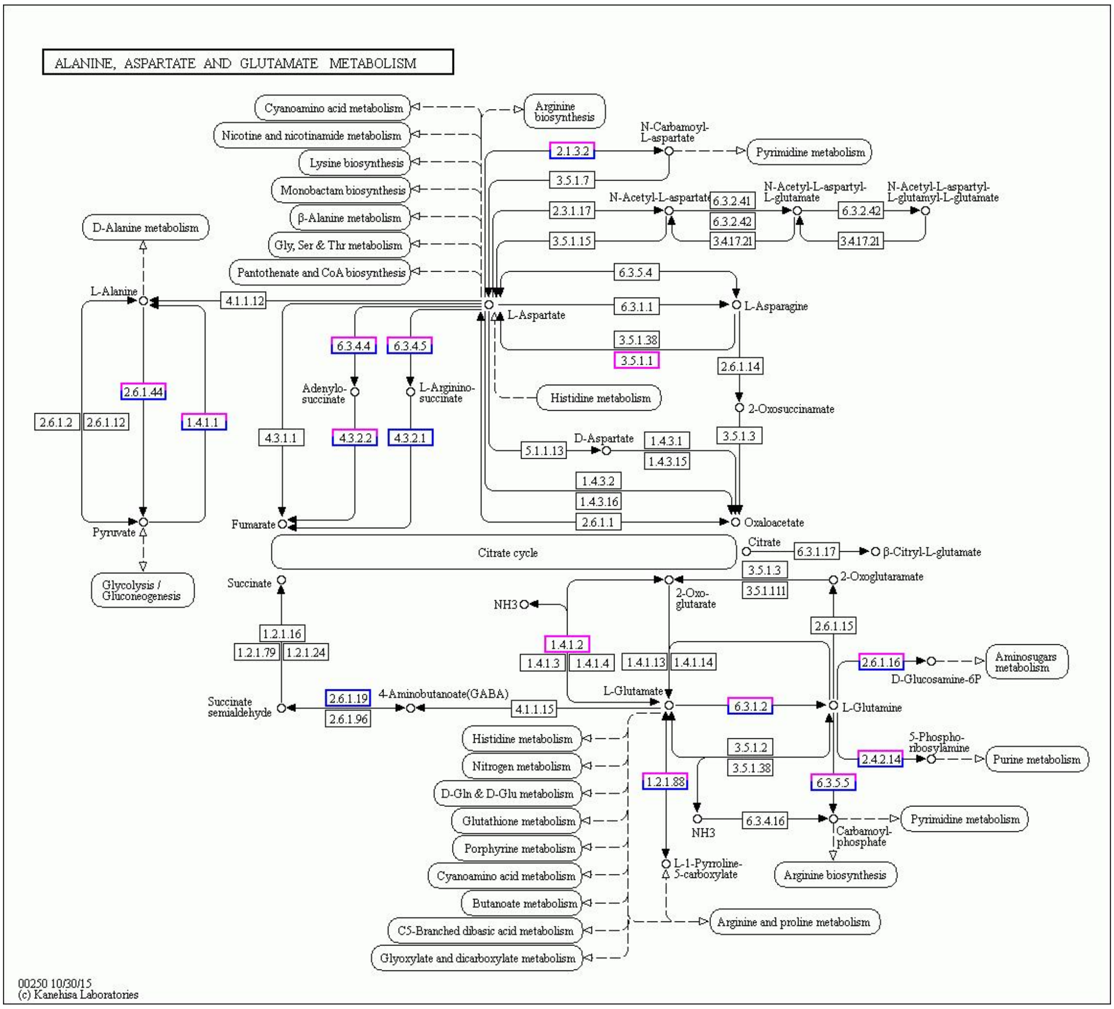

Fig. S10 Alanine, aspartate and glutamate metabolism pathway of S. aureus SCVs correlation analysis from KEGG website. The blue boxes indicate differentially expressed genes (DEGs) and pink boxes indicate differentially expressed proteins (DEPs). The process of L-aspartate converted into adenylosuccinate and L-argininosuccinate respectively and further conversion into fumarate was inhibited (6.3.4.4, 6.3.4.5, 4.3.2.2 and 4.3.2.1 all downregulated).

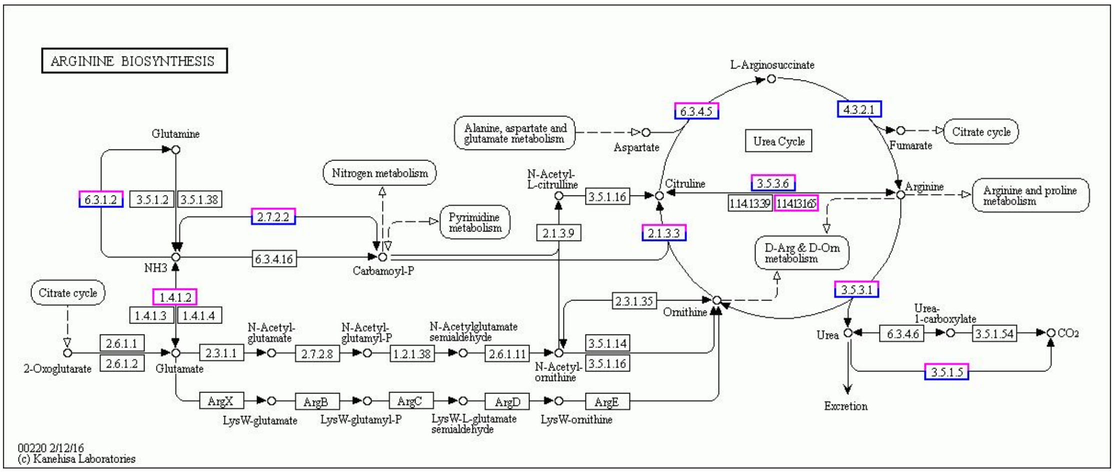

Fig. S11 Arginine biosynthesis pathway of S. aureus SCVs correlation analysis from KEGG website. The blue boxes indicate differentially expressed genes (DEGs) and pink boxes indicate differentially expressed proteins (DEPs). The process of citruline converted to L-arginosuccinate then produced fumarate was suppressed (6.3.4.5 and 4.3.2.1 all downregulated).

Supplements 1-6 titles

Supplement 1 IE2-VS-IE1_transcriptome DEGs

Supplement 2 Transcriptome pathways and genes

Supplement 3 IE2-VS-IE1_proteome DEPs

Supplement 4 Proteome pathways and proteins

Supplement 5 IE2-VS-IE1_correlation DEGs and DEPs

Supplement 6 Correlation pathways
